# COVID-19 Readmission Is Highest Among Refugees in Denmark

**DOI:** 10.3390/ijerph22030367

**Published:** 2025-03-03

**Authors:** Amar Ali Moussa, Marwa Mohammad, Andreas Halgreen Eiset, Signe Freja Storgaard, Christian Wejse

**Affiliations:** 1 The Research Unit for Global Health, Department of Public Health, Aarhus University, 8000 Aarhus, Denmark; marwa.95@live.dk (M.M.);; 2 Department of Infectious Diseases, Aarhus University Hospital, 8000 Aarhus, Denmark

**Keywords:** COVID-19, migrant health, refugee health, readmission, COVID-19 status at admission

## Abstract

Vulnerable groups, including certain immigrant populations, have faced higher COVID-19 incidence rates in several countries. This study addresses the gap in knowledge regarding disease severity and readmission odds among refugees, other immigrant groups, and native Danes. Using clinical data from 159 COVID-19-positive patients admitted to hospitals in the Central Denmark Region in 2020, this cross-sectional analysis compared clinical parameters at admission and 30-day readmission odds. The findings revealed no significant differences in clinical status upon admission between groups. Refugees (51.8%) and Others (41.7%) had fewer comorbidities than native Danes (61.2%). Native Danes were more frequently categorized with the highest Charlson Comorbidity Index (CCI) scores. Readmission prevalence was highest among Refugees (23.1%), followed by native Danes (17.0%) and Others (8.3%). After adjusting for age, sex, and CCI, Refugees had a readmission odds ratio (OR) of 1.88 (95% CI, 0.61–5.74) and Others had an OR of 0.61 (95% CI, 0.07–5.41) for readmission compared to native Danes, although this was not statistically significant. This study’s significance lies in highlighting the distinct healthcare challenges faced by refugees during the pandemic. Its findings are beneficial for public health policymakers and healthcare professionals seeking to reduce readmission risks and improve COVID-19 outcomes for immigrant populations.

## 1. Introduction

Coronavirus Disease 2019 (COVID-19) is a respiratory infection that caused a global pandemic, impacting all layers of society. Initially, in Denmark, COVID-19 cases were primarily found among well-educated and globalized individuals, but the infection quickly spread to less privileged groups [1]. Throughout the pandemic, Western countries, including Denmark, observed higher COVID-19 incidence rates among certain immigrant groups compared to the host population [1,2,3,4]. In 2019, 10% of the Danish population consisted of immigrants, with 58% from non-Western countries [5]. A report from the Danish Health Authority showed that immigrants from Iran, Turkey, Pakistan, Somalia, Lebanon, the former Yugoslavia, and Iraq experienced a higher burden of disease compared to native Danes. This report also found a higher incidence of various diseases, including diabetes, chronic respiratory diseases, and heart diseases [6]. These comorbidities have been identified as risk factors for severe COVID-19 outcomes [7,8] raising concerns for immigrant groups with high COVID-19 incidence and comorbidities. Moreover, immigrants and refugees are also at high risk of other infectious diseases, such as HIV [9], tuberculosis [9], HPV [9], and hepatitis [9].

Certain immigrant groups, including those from Iran, Turkey, Pakistan, the former Yugoslavia, Poland, Sri Lanka, Lebanon, Syria, Afghanistan, and Iraq, are more likely to have lower socioeconomic status (SES) in terms of poorer education, economic conditions, and occupational parameters compared to native Danes [5]. SES is known to influence health status, with low SES generally having a negative effect. It is associated with a higher incidence of somatic diseases, psychiatric conditions, and reduced life expectancy. SES also impacts housing conditions and employment type, factors that can affect the transmission of COVID-19 [1,5,6,10].

Not only are immigrants at higher risk of COVID-19 transmission, but studies have also shown that their COVID-19 outcomes are more severe compared to host populations [2,4]. Certain vital parameters, biomarkers, and clinical factors have been used as predictors for severe COVID-19 outcomes, including C-reactive protein (CRP), lactate dehydrogenase (LDH), D-dimer, body temperature, and peripheral oxygen saturation (SAT) [11,12,13,14,15,16,17]. Additionally, older age, male sex, smoking, high BMI, and comorbidities have emerged as important risk factors for adverse outcomes, such as readmission [7,17,18,19,20,21]. Readmitted patients may be at increased risk of hypoxia and death [22].

Lower socioeconomic status (SES), higher incidence of comorbidities, and increased COVID-19 rates among immigrants raise concerns that they may be a vulnerable group regarding adverse outcomes from COVID-19. Data on immigrants with COVID-19 in Denmark are limited. Therefore, it is necessary to clarify the characteristics and outcomes of COVID-19 cases in the Danish population, with a focus on the role of immigrant status and more targeted public health interventions for immigrants [23]. In this study, the hypothesis is that no difference in COVID-19 readmission exists within the study population after adjustment for certain variables. The aim of this study is to examine whether there is a clinical difference in the severity of COVID-19 at the time of hospital admission based on patients’ immigrant status. Moreover, the study will investigate whether a difference in COVID-19 readmission exists among patients depending on their immigrant status.

## 2. Materials and Methods

### 2.1. Study Design and Study Population

This cross-sectional study was based on clinical data from patients admitted to hospitals in the Central Denmark Region (including Aarhus University Hospital, the Regional Hospital in Horsens, the Regional Hospital in Viborg, and the Regional Hospital in Goedstrup) due to COVID-19 between 1 January 2020, and 4 November 2020. COVID-19 diagnosis was confirmed based on a positive real-time reverse-transcriptase-polymerase-chain reaction (RT-PCR) test from a pharyngeal swab.

The inclusion criteria for this study required participants to have a confirmed positive COVID-19 test, be above 18 years of age, and have experienced at least one prior hospital discharge following COVID-19 treatment. All eligible patients within the study period who provided informed consent for inclusion in the COVID-19 database at Aarhus University Hospital were included. Informed written consent was documented and stored within the electronic patient journal. A total of 159 patients met these criteria, though five were excluded due to mortality during their initial COVID-19 admission.

**Figure 1:**
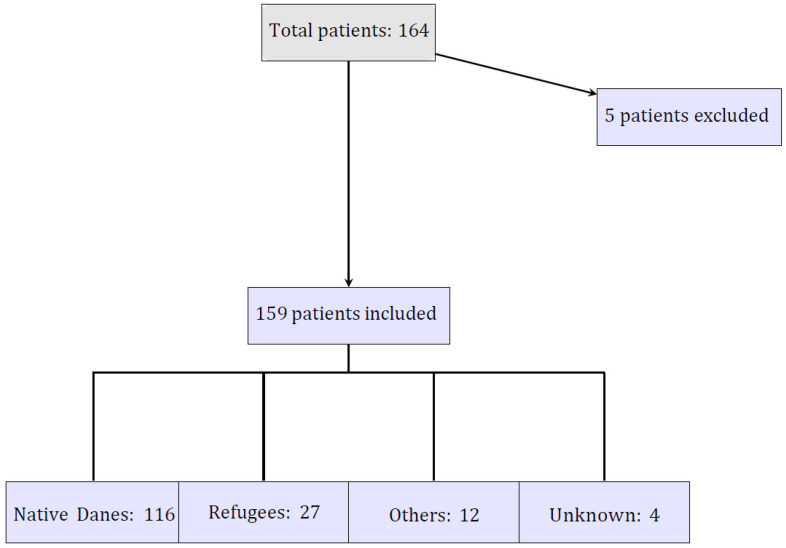
Flowchart of study population.

The study population was categorized into three groups based on their country of origin: asylum-generating countries, other countries of origin, and native Danes. Asylum-generating countries included Syria, Somalia, Afghanistan, Iraq, and individuals with Palestinian and Kurdish roots [1,5,24,25,26]. Those from asylum-generating countries were assumed to hold a Danish residence permit through asylum application and were therefore classified as “Refugees”. Immigrants from countries such as Turkey, Iran, the former Yugoslavia, Poland, England, Sri Lanka, and Comoros were assumed to hold Danish residence permits for purposes such as work, education, or family reunification and were classified as “Others” [5,25,27]. The “native Danes” group consisted of individuals of Danish ethnicity and served as the reference group [6]. The study population is illustrated in Figure 1.

### 2.2. Data Collection and Variables

Patient data, including age, sex, smoking status, BMI, comorbidities, immigrant status, clinical parameters, biomarkers, and readmission status, were obtained through a quantitative survey of COVID-19 patients admitted to hospital. The survey data were extracted entirely from the electronic patient journal using the patients’ unique Danish Personal Identification number (CPR number). Patient sex was obtained through the CPR number. All data were collected in the Research Electronic Data Capture (REDCap 14.5.36) system for patients who met the inclusion criteria during the study period. Exposure, stratified by immigrant status, was identified by searching patient records using key terms such as country of origin, social background, and translation needs.

The outcomes of interest were COVID-19 readmission within 30 days and clinical status at admission, which was characterized by parameters such as temperature, SAT, CRP, D-dimer, and LDH.

The Charlson Comorbidity Index (CCI) was used to assess comorbidity burden rather than examining the effect of specific comorbidities. The CCI predicts 10-year survival in patients with multiple comorbidities using a scoring system based on the severity of each comorbidity [28,29,30]. The CCI has been used in several COVID-19 studies to measure comorbidity burden [21,31,32]. Additionally, the CCI was used as a predictor for severe outcomes such as readmission. The CCI score is calculated based on the presence and severity of comorbidities, with each condition assigned a score ranging from 1 to 6 points [28]. Comorbidities considered include myocardial infarction, heart failure, peripheral vascular disease, cerebrovascular disease, dementia, chronic pulmonary disease, rheumatic disease, peptic ulcer disease, liver disease, diabetes (with or without complications), hemiplegia, renal disease, malignancies (excluding skin neoplasms), metastatic solid tumors, and AIDS/HIV. Other comorbidities were not included in this analysis. In this study, the CCI was not adjusted for age, as age was treated as an independent variable in the adjusted model.

### 2.3. Statistical Analysis

The statistical analysis included 159 patients and was performed using the statistical software package StataSE 16.1. All data management was conducted through a secure Virtual Desktop Infrastructure (VDI) program. Logistic regression was used for the statistical analysis, with immigrant status as the exposure variable and readmission within 30 days as the outcome event. The analysis provided an odds ratio (OR) along with a 95% confidence interval (CI). A crude model and two adjusted models were created. All reported *p*-values were two-sided, with *p* < 0.05 considered statistically significant. Model 1 was adjusted for age and sex, while Model 2 was additionally adjusted for the CCI. These variables were prioritized based on current evidence regarding their impact on the severity of COVID-19 outcomes. Due to the small sample size and the limited number of readmitted patients, a logistic regression model incorporating additional variables could not be performed because of constraints on degrees of freedom.

## 3. Results

Table 1 presents the baseline characteristics of native Danes, Refugees, and Others and a column for individuals with unknown immigrant status or missing data. The study included 116 native Danes, 27 Refugees, 12 Others, and 4 individuals with missing country of origin. The median age was lower among Refugees and Others compared to native Danes, with values of 54 [IQR: 39–61] years, 53 [IQR: 45–62] years, and 67 [IQR: 52–75] years, respectively. A total of 43.4% of the study participants were female. All groups exhibited a median body temperature that was either subfebrile or febrile at the time of admission. C-reactive protein (CRP) levels were elevated in all groups at admission. The three groups had similar median CCI scores. In total, 27 of the 159 patients (17.5%) were readmitted due to COVID-19. The highest readmission rate was observed among Refugees, at 23.1%, compared to 8.3% for Others and 17.0% for native Danes.

Table 2 illustrates the distribution of Charlson Comorbidity Index (CCI) scores across the different groups. The table shows that 48.2% of Refugees, 58.3% of Others, and 38.8% of native Danes had no comorbidities. Generally, the distribution for Refugees and Others was predominantly in the 0–2 point range, with a lower percentage of these groups scoring 3–7 points compared to native Danes. There were no missing data regarding comorbidities, but four individuals had an unknown immigrant status. Among them, two had 0 CCI points, one had 2 CCI points, and one had 6 CCI points.

Table 3 presents the OR with (95% CI) for COVID-19 readmission across the groups. The crude OR for readmission was 1.47 (95% CI, 0.52–4.14) for Refugees and 0.44 (95% CI, 0.05-3.65) for Others compared to native Danes. In Model 1, the OR for readmission was 1.89 (95% CI, 0.62–5.79) for Refugees and 0.63 (95% CI, 0.07–5.47) for Others. In Model 2, the OR for readmission was 1.88 (95% CI, 0.61–5.74) for Refugees and 0.61 (95% CI, 0.07–5.41) for Others, indicating that Refugees had the highest odds of readmission.

## 4. Discussion

A difference in status at admission between Refugees, Others, and native Danes was not observed based on certain clinical parameters and biomarkers. Overall, the results indicated that Refugees and Others had fewer comorbidities compared to native Danes, while Refugees had the highest adjusted OR for readmission. This could be explained by the fact that native Danes were the oldest group, and the risk of comorbidities increases with age [33,34].

At the time of the study, COVID-19 was a relatively new infection, and the literature surrounding the disease was still limited. It remains unclear which clinical parameters, biomarkers, and comorbidities are the primary risk factors for severe COVID-19 outcomes [11].

Several factors have been suggested to explain the higher COVID-19 incidence among vulnerable groups compared to host populations. One of the main reasons is housing and family conditions. Immigrants generally live with more people and multiple generations in smaller living spaces compared to native Danes [1]. This can complicate isolation and make elderly immigrants more vulnerable to COVID-19 transmission. Additionally, immigrants are overrepresented in occupations with a high risk of exposure to COVID-19 and close physical proximity to others, such as transportation, cleaning services, hotels, restaurants, health services, and social services. These sectors are closely linked to COVID-19 cases in Denmark, according to the Danish State Serum Institute [35]. Furthermore, national campaigns providing information on COVID-19 have had limited effectiveness among some immigrant groups due to language barriers and lack of education. Despite written translations in several languages from the Danish Health Authority, some immigrants, particularly the elderly, have limited literacy skills in their native language [1].

Several studies have indicated that race is not an independent risk factor for severe COVID-19 outcomes [1,20,35,36,37,38,39,40]. However, variations in hospitalization and readmission rates reveal complex intersections between race, socioeconomic status (SES), and healthcare access. An American study on 106,543 post-discharge patients over two months found a 9% readmission rate, with White individuals more likely to be readmitted when adjusted for COVID-19 risk factors [36]. This contrasts with findings linking higher hospitalization rates to racial minority groups, highlighting potential differences in healthcare access, health-seeking behaviors, or regional disparities.

A Korean study reported a 4.3% readmission rate among 7590 patients, with those receiving medical benefits at greater risk of readmission, underscoring the influence of SES on COVID-19 outcomes [37,38]. This aligns with broader findings highlighting how socioeconomic factors, such as access to healthcare and living conditions, often outweigh racial or ethnic differences in determining health outcomes. Similarly, a study found that while Hispanic patients had higher hospitalization rates, mortality was higher among White patients, likely due to their older age distribution [39]. This suggests that while hospitalization rates may disproportionately affect minority groups, age-related vulnerabilities remain a critical factor in severe outcomes. Moreover, Black patients demonstrated higher positivity and hospitalization rates but no increased risk of ICU admission [20]. This apparent disconnect between hospitalization and ICU admission rates illustrates how healthcare access disparities may manifest differently across racial groups. Moreover, an American retrospective cohort study highlighted that racial disparities in COVID-19 readmission risks were not biologically driven but linked to structural inequities, such as access to healthcare and socioeconomic disadvantages [41].

Overall, these findings converge on the critical insight that structural inequities, rather than inherent biological differences, shape COVID-19 outcomes. Practically, these findings emphasize the necessity of targeted public health policies that address the multi-faceted risk factors affecting vulnerable populations, particularly ethnic minorities. By incorporating these insights into pandemic preparedness and response strategies, healthcare systems can better mitigate the disproportionate impact on marginalized communities, fostering a more inclusive and equitable healthcare environment for all.

Hospital readmissions can negatively impact patient outcomes and increase hospital expenses. While the reasons for hospital readmissions are not fully understood, poor care coordination after discharge and inadequate follow-up care have been suggested as key factors [42]. One study indicates that patients with lower SES were less likely to discuss health concerns with healthcare professionals after discharge. Additionally, the study found that male patients were less likely to understand self-care instructions after discharge compared to females [42]. A delayed response to signs of worsening COVID-19 may explain why some patients require readmission, contributing to longer stays. Shorter initial admissions could also lead to readmission due to inadequate treatment during the first hospitalization [43].

COVID-19 restrictions may also influence the course of admission and readmission. For instance, during the pandemic, hospitals enforced strict visiting restrictions [44]. This could have been particularly challenging for immigrant patients, who are often accustomed to having multiple family members present [1]. Furthermore, language barriers between patients and healthcare professionals might be exacerbated by these restrictions. An American study found that patients who received interpretation services at admission and/or discharge were less likely to be readmitted within 30 days compared to those who did not receive such services [45]. In Denmark, free translation services are only available to select patients, often resulting in relatives acting as translators [46,47].

One of the limitations of this study is the small sample size, which may have limited the estimates of the logistic regression. The small sample size could have also affected the differences in status at admission between the groups, particularly in variables with a high number of missing data [48,49]. Additionally, selection bias is a limitation in this study, as the statistical analysis only included complete cases, and thus, missing data were not taken into account. Furthermore, the logistic regression only adjusted for certain variables, which were prioritized based on current evidence regarding factors influencing the severity of COVID-19 outcomes. However, interactions between these variables were not considered in the logistic regression analysis, meaning that the results were not adjusted for the potential modifying effects of these interactions.

A strength of this study is that it included patients from an entire region of Denmark, the Central Denmark Region, which makes the results more representative of all cases in Denmark. Another strength is the grouping of the study population, which accounts for differences in SES depending on immigrant status [5]. This approach provides a more valid result regarding how immigrant status affects readmission.

In a cross-sectional study like this, causal associations cannot be established. Instead, the study highlights associations. In this study, the primary focus was on examining the associations between our exposure—immigrant status—and the outcome—COVID-19 readmission within 30 days.

## 5. Conclusions

In summary, no notable differences in status at admission between Refugees, Others, and native Danes were observed based on body temperature, SAT, CRP, D-dimer, and LDH. Overall, Refugees and Others had fewer comorbidities than native Danes. The results showed that 27 (17.5%) of 159 patients were readmitted in relation to COVID-19, with Refugees having the highest prevalence among the groups. A difference in the OR for readmission between the groups was observed after adjustment for certain variables, with Refugees having the highest adjusted OR. However, these ORs were not statistically significant.

While COVID-19 has been extensively studied, there remains a lack of data in Denmark regarding the association between immigrant status and readmission rates. In Denmark, the COVID-19 pandemic ignited considerable debate about ethnic minorities and their role in the pandemic, underscoring the importance of investigating this topic to develop a more nuanced understanding of these dynamics. Further research is required to better understand the relationship between immigrant status and both admission status and readmission rates among COVID-19 patients.

## Figures and Tables

**Table 1:** Baseline characteristics of COVID-19 patients.

Characteristics	All	Native Danes	Refugees	Others	Unknown *
Total *^a^*	159 (100%)	116 (73%)	27 (17%)	12 (7.5%)	4
Age *^b^*, years	59 [50–73]	67 [52–75]	54 [39–61]	53 [45–62]	2
Sex *^a^*, female	69 (43.4%)	51 (44%)	11 (40.7%)	6 (50%)	0
Smoking *^a^*					
Never	74 (54.8%)	51 (50.8%)	13 (65.0%)	7 (77.8%)	24 *^c^*
Former	52 (38.5%)	45 (44.1%)	5 (25.0%)	1 (11.1%)	
Current	9 (6.7%)	6 (5.9%)	2 (10.0%)	1 (11.1%)	
BMI *^b^*, kg/m^2^	26.7 [24.2–30.4]	26.5 [24.2–30.3]	26.7 [24.6–34.2]	29.4 [24.0–35.2]	22
Clinical parameters **					
Body temperature *^b^*^,*d*^	38.3 [37.7–39.1]	38.3 [37.7–39.2]	38.5 [37.5–39.1]	37.8 [37.3–39.2]	10
SAT *^b^*, %	96 [94–97]	95 [94–97]	97 [95–99]	97 [96–99]	10
Clinical biomarkers **					
CRP *^b^*, mg/L	42 [18–91]	43 [18–109]	35 [24–83]	48 [4–86]	38
D-dimer *^b^*, mg/L (FEU)	0.7 [0.4–1.1]	0.7 [0.5–1.3]	0.6 [0.3–1.1]	0.8 [0.4–3.8]	102
LDH *^b^*, U/L	239 [173–348]	247 [187–357]	190 [158–269]	284 [166–357]	95
CCI *^b^*	1 [0–2]	1 [0–2]	1 [0–2]	0 [0–1]	0
COVID-19 readmission *^a^*		19 (17.0%)	6 (23.1%)	1 (8.3%)	
Yes	27 (17.5%)				
No	127 (82.5%)	93 (83.0%)	20 (76.9%)	11 (91.7%)	5

*a*: Total number (%), *b*: Median [interquartile range], *c*: Total number of missing data regarding smoking, *d*: Degrees Celsius, * Total number of missing data, and ** The clinical parameters and biomarkers obtained at the onset of admission. Abbreviations: COVID-19, Corona Virus Disease 2019; BMI, Body Mass Index; CRP, C-Reactive Protein; FEU, Fibrinogen-Equivalent Units; LDH, Lactate Dehydrogenase; SAT, Saturation; CCI, Charlson Comorbidity Index.

**Table 2:** Charlson Comorbidity Index *.

CCI	All (*n* = 159)	Native Danes (*n* = 116)	Refugees (*n* = 27)	Others (*n* = 12)
0	67 (42.1%)	45 (38.8%)	13 (48.2%)	7 (58.3%)
1	42 (26.4%)	32 (27.6%)	7 (25.9%)	3 (25.0%)
2	33 (20.8%)	25 (21.6%)	6 (22.2%)	1 (8.3%)
3	6 (3.8%)	6 (5.2%)	0	0
4	5 (3.1%)	3 (2.6%)	1 (3.7%)	1 (8.3%)
5	2 (1.3%)	2 (1.7%)	0	0
6	2 (1.3%)	1 (0.9%)	0	0
7	2 (1.3%)	2 (1.7%)	0	0

* Total number (%).

**Table 3:** Odds ratios (ORs) for readmission of COVID-19 patients.

			Model 1 *:		Model 2 **:	
Group	Crude OR (95% CI)	*p*-Value	Adjusted OR (95% CI)	*p*-Value	Adjusted OR (95% CI)	*p*-Value
Native Danes	1.0	-	1.0	-	1.0	-
Refugees	1.47 (0.52–4.14)	0.47	1.89 (0.62–5.79)	0.26	1.88 (0.61–5.74)	0.27
Others	0.44 (0.05–3.65)	0.45	0.63 (0.07–5.47)	0.67	0.61 (0.07–5.41)	0.66

* Model 1: Adjusted for age and sex; ** Model 2: Adjusted for age, sex, and CCI.

## Data Availability

The data supporting the findings of this study is not available due to privacy restrictions.
